# Genome-Wide Methylation Profiling in 229 Patients With Crohn’s Disease Requiring Intestinal Resection: Epigenetic Analysis of the Trial of Prevention of Post-operative Crohn’s Disease (TOPPIC)

**DOI:** 10.1016/j.jcmgh.2023.06.001

**Published:** 2023-06-17

**Authors:** Nicholas T. Ventham, Nicholas A. Kennedy, Rahul Kalla, Alex T. Adams, Alexandra Noble, Holly Ennis, TOPPIC Study Group, Ian Arnott, Aiden Cahill, Malcolm Smith, Tariq Ahmad, Sreedhar Subramanian, Simon Travis, John Morris, John Hamlin, Anjan Dhar, Chuka Nwokolo, Cathryn Edwards, Tom Creed, Stuart Bloom, Mohamed Yousif, Linzi Thomas, Simon Campbell, Stephen J. Lewis, Shaji Sebastian, Sandip Sen, Simon Lal, Chris Hawkey, Charles Murray, Fraser Cummings, Jason Goh, James O. Lindsay, Naila Arebi, Lindsay Potts, Aileen J. McKinley, John M. Thomson, John A. Todd, Mhairi Collie, Ashley Mowat, Daniel R. Gaya, Jack Winter, Graham D. Naismith, Holly Ennis, Catriona Keerie, Steff Lewis, Robin J. Prescott, IBD-BIOM Consortium, Gordan Lauc, Harry Campbell, Dermot P.B. McGovern, Vito Annese, Vlatka Zoldoš, Iain K. Permberton, Manfred Wuhrer, Daniel Kolarich, Daryl L. Fernandes, Evropi Theorodorou, Victoria Merrick Daniel I. Spencer, Richard A. Gardner, Ray Doran, Archana Shubhakar, Ray Boyapati, Igor Rudan, Paolo Lionetti, Irena Trbojević Akmačić, Jasminka Krištić, Frano Vuč ković, Jerko Štambuk, Mislav Novokmet, Maja Pučić-Baković, Olga Gornik, Angelo Andriulli, Laura Cantoro, Giancarlo Sturniolo, Gionata Fiorino, Natalia Manetti, Anna Latiano, Anna Kohn, Renata D’Inca`, Silvio Danese, Ian D. Arnott, Colin L. Noble, Charlie W. Lees, Alan G. Shand, Gwo-Tzer Ho, Lee Murphy, Jude Gibson, Louise Evenden, Nicola Wrobel, Tamara Gilchrist, Angie Fawkes, Guinevere S.M. Kammeijer, Florent Clerc, Noortje de Haan, Aleksandar Vojta, Ivana Samaržija, Dora Markulin, Marija Klasić, Paula Dobrinić, Yurii Aulchenko, Tim van den Heuve, Daisy Jonkers, Marieke Pierik, Craig Mowat, Malcolm G. Dunlop, Jack Satsangi

**Affiliations:** Centre for Genomic and Experimental Medicine, The University of Edinburgh, Edinburgh, Midlothian, United Kingdom

**Keywords:** Crohn's disease, Surgery, DNA methylation, Epigenetics, Inflammatory bowel disease, Aging

## Abstract

**Background & Aims:**

DNA methylation alterations may provide important insights into gene-environment interaction in cancer, aging, and complex diseases, such as inflammatory bowel disease (IBD). We aim first to determine whether the circulating DNA methylome in patients requiring surgery may predict Crohn’s disease (CD) recurrence following intestinal resection; and second to compare the circulating methylome seen in patients with established CD with that we had reported in a series of inception cohorts.

**Methods:**

TOPPIC was a placebo-controlled, randomized controlled trial of 6-mercaptopurine at 29 UK centers in patients with CD undergoing ileocolic resection between 2008 and 2012. Genomic DNA was extracted from whole blood samples from 229 of the 240 patients taken before intestinal surgery and analyzed using 450KHumanMethylation and Infinium Omni Express Exome arrays (Illumina, San Diego, CA). Coprimary objectives were to determine whether methylation alterations may predict clinical disease recurrence; and to assess whether the epigenetic alterations previously reported in newly diagnosed IBD were present in the patients with CD recruited into the TOPPIC study. Differential methylation and variance analysis was performed comparing patients with and without clinical evidence of recurrence. Secondary analyses included investigation of methylation associations with smoking, genotype (MeQTLs), and chronologic age. Validation of our previously published case-control observation of the methylome was performed using historical control data (CD, n = 123; Control, n = 198).

**Results:**

CD recurrence in patients following surgery is associated with 5 differentially methylated positions (Holm *P* < .05), including probes mapping to *WHSC1* (*P* = 4.1 × 10^-9^, Holm *P* = .002) and *EFNA3* (*P* = 4.9 × 10^-8^, Holm *P* = .02). Five differentially variable positions are demonstrated in the group of patients with evidence of disease recurrence including a probe mapping to *MAD1L1* (*P* = 6.4 × 10^-5^). DNA methylation clock analyses demonstrated significant age acceleration in CD compared with control subjects (GrimAge + 2 years; 95% confidence interval, 1.2–2.7 years), with some evidence for accelerated aging in patients with CD with disease recurrence following surgery (GrimAge +1.04 years; 95% confidence interval, -0.04 to 2.22). Significant methylation differences between CD cases and control subjects were seen by comparing this cohort in conjunction with previously published control data, including validation of our previously described differentially methylated positions (*RPS6KA2 P* = 1.2 × 10^-19^, *SBNO2* = 1.2 × 10^-11^) and regions (*TXK* [false discovery rate, *P* = 3.6 × 10^-14^], *WRAP73* [false discovery rate, *P* = 1.9 × 10^-9^], *VMP1* [false discovery rate, *P* = 1.7 × 10^-7^], and *ITGB2* [false discovery rate, *P* = 1.4 × 10^-7^]).

**Conclusions:**

We demonstrate differential methylation and differentially variable methylation in patients developing clinical recurrence within 3 years of surgery. Moreover, we report replication of the CD-associated methylome, previously characterized only in adult and pediatric inception cohorts, in patients with medically refractory disease needing surgery.

## Graphical abstract

**Figure:**
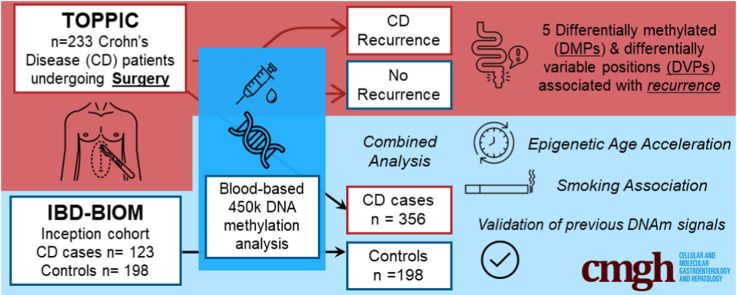

**Summary**

Detailed study of the circulating DNA methylome in adults with new and established Crohn’s disease undergoing surgery within a large randomized controlled trial. Methylation alterations are observed in patients with post-operative disease recurrence.

DNA methylation is an important epigenetic mechanism that associates with alteration in gene expression with no underlying change in the genetic code. DNA methylation changes have been implicated in cancer; aging^1^, ^2^, ^3^, ^4^; and many complex diseases, including inflammatory bowel disease (IBD).^5^^,^^6^

In our original studies, we described the circulating “methylome” in patients with IBD and control subjects,^7^^,^^8^ including in a large inception cohort of newly diagnosed patients.^9^ These methylation differences across the genome in peripheral blood leucocyte DNA correlate with known clinical parameters of inflammation, but importantly relate to underlying genotype. A key potential importance of DNA methylation changes relates to an association with alteration of gene expression. We were able to demonstrate the appropriate inverse relationship between methylation and gene expression, in a cell-specific manner in separated circulating leukocytes.^9^ Most recently, we have provided strong replication of these methylation signals in a large inception cohort of patients with IBD recruited across Northern Europe, and replication of some signals in Southern Europe.^10^

Although genome-wide methylation differences have been demonstrated between IBD cases and control subjects, identifying methylomic differences between IBD subphenotypes is more nuanced. Multiomic data have also been used to prognosticate in IBD, attempting to delineate patients at risk of severe disease phenotype requiring surgery or more intensive drug regimens.^11^, ^12^, ^13^, ^14^, ^15^ Using an unsupervised clustering method in our index study of an inception cohort of patients with Crohn’s disease (CD) and ulcerative colitis, we identified groups of patients potentially at higher risk of surgery or treatment escalation.^9^ In a large treatment-naive inception cohort in Europe, we identified 3 methylation probes (*TAP1*, *TESPA1*, *RPTOR*) that associated with the need for treatment escalation to biologic agents or surgery.^10^

Patients with CD have a high lifetime risk of surgery for refractory or complicated disease. Approximately half of patients undergo surgery within 10 years of diagnosis;^16^ however, with the introduction of newer biologic treatment, surgery rates seem to be falling.^17^ The TOPPIC trial sought to determine the efficacy of 6-mercaptopurine (6-MP) in prevention of the recurrence of disease following ileocolic resection.^18^ Two-hundred and forty patients were randomized across 29 UK centers to receive 6-MP or placebo following ileocolic resection for CD. The primary end point was a composite clinical end point that included an increase in Crohn’s disease activity index score, requirement for treatment escalation, or further surgery. The trial showed a modest benefit with 6-MP treatment versus placebo for the primary clinical end point (hazard ratio, 0.54; 95% confidence interval [CI], 0.27–1.06). There was a more pronounced benefit for 6-MP for smokers (hazard ratio, 0.13; 95% CI, 0.04–0.46).^18^

The coprimary aims of the present study were to determine whether circulating DNA methylation differences in patients before surgery differ between patients with and without evidence of clinical or endoscopic recurrence following surgical resection; and to extend our observations of methylation alterations made in inception cohorts of newly diagnosed patients by studying an independent cohort of patients with established CD requiring surgery (Figure 1).

## Results

### Participants, Demographics, Data Processing, and Quality Control

There were 233 TOPPIC samples available for analysis with no samples failing quality control. Patient demographic information is presented for TOPPIC participants in Table 1. Data processing procedures demonstrated visually improved characteristics on density plots Figure 2*A–D*) and multidimensional scaling (MDS) plots (Figure 2*E–G*). After filtering, 429,944 probes were available for analysis. No samples failed sex check (Figure 2*F*). QQ plots, Lambda values, and clustering of cohorts on MDS plots improved following combat correction (for both array number and intra-array position; Figure 3). Four TOPPIC patients had missing outcome data and were excluded from disease recurrence analyses.

**Table 1:** Patient Demographics of Patients Included From the TOPPIC Trial, With and Without Clinical Recurrence^a^

	Clinical recurrence (n = 40)	No recurrence (n = 189)	*P* value
Female, n (*%*)	26 (65.0)	115 (60.8)	.9
Age, *y*, median (IQR)	32.2 (27.8–41.0)	40.0 (29.0–49.8)	.02
Baseline CDAI (IQR)	124.5 (73.7–223.0)	112.0 (64.5–164.0)	.2
Disease location, n (*%*)			0.7
L1 ileal	14 (35.0)	74 (39.2)	
L3 ileocolonic	26 (65.0)	115 (60.8)	
Disease behavior, n (*%*)			0.8
Inflammatory B1	17 (42.5)	73 (38.9)	
Stricturing B2	17 (42.5)	89 (47.3)	
Penetrating B3	6 (15.0)	26 (13.8)	
Previous infliximab, n (*%*)	7 (17.5)	29 (15.7) (3missing)	1
Previous azathioprine, n (*%*)	22 (55)	102 (54.3)	1
Previous surgery, n (*%*)	9 (22.5)	62 (32.8)	.3
Current smoker, n (*%*)	14 (35)	40 (21.2)	.1
Biochemistry median (IQR)			
CRP, *mg/L*	3.7 (3–6)	4 (3–7)	.7
ESR	13 (6–22) (3 missing)	12 (5–19) (50 missing)	.4
Albumin	42 (41–46)	43 (40–45)	.8
White cell count	7.1 (5.5–8.5)	6.6 (5.5–8.1)	.4

NOTE. Results are presented as median, interquartile range. Nonparametric statistics are used to compare groups, chi-square for categorical data and Wilcox rank sum test for continuous data.

CDAI, Crohn’s disease activity index; CRP, C-reactive protein; ESR, erythrocyte sedimentation rate; IQR, interquartile range;

a Defined as increase in CDAI of more than 150 and an increase of 100 points from baseline measurement and institution of immunosuppressive treatment or further surgery.

**Figure 1:**
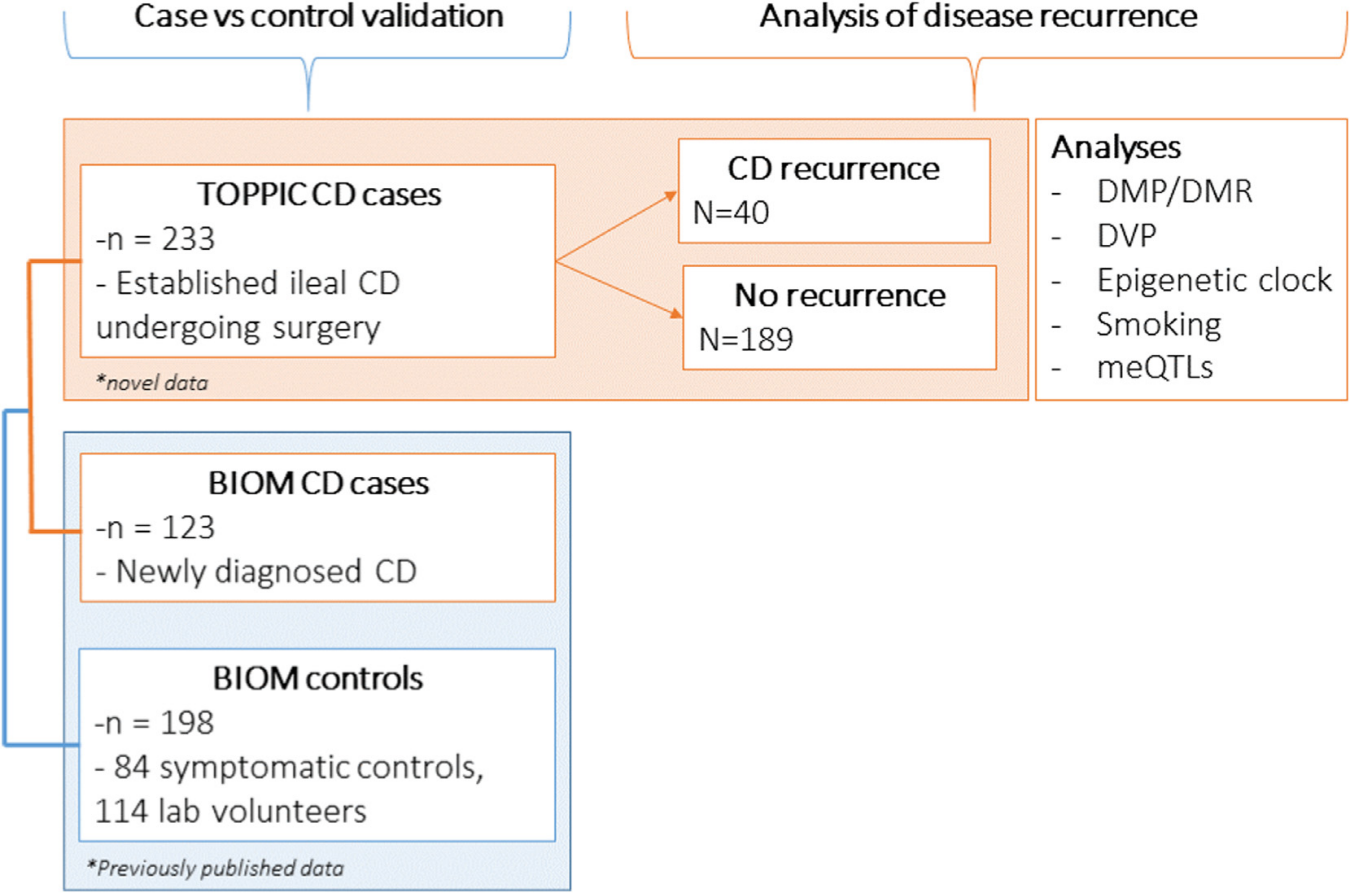
**Flowchart of cohorts and analyses**.

**Figure 2:**
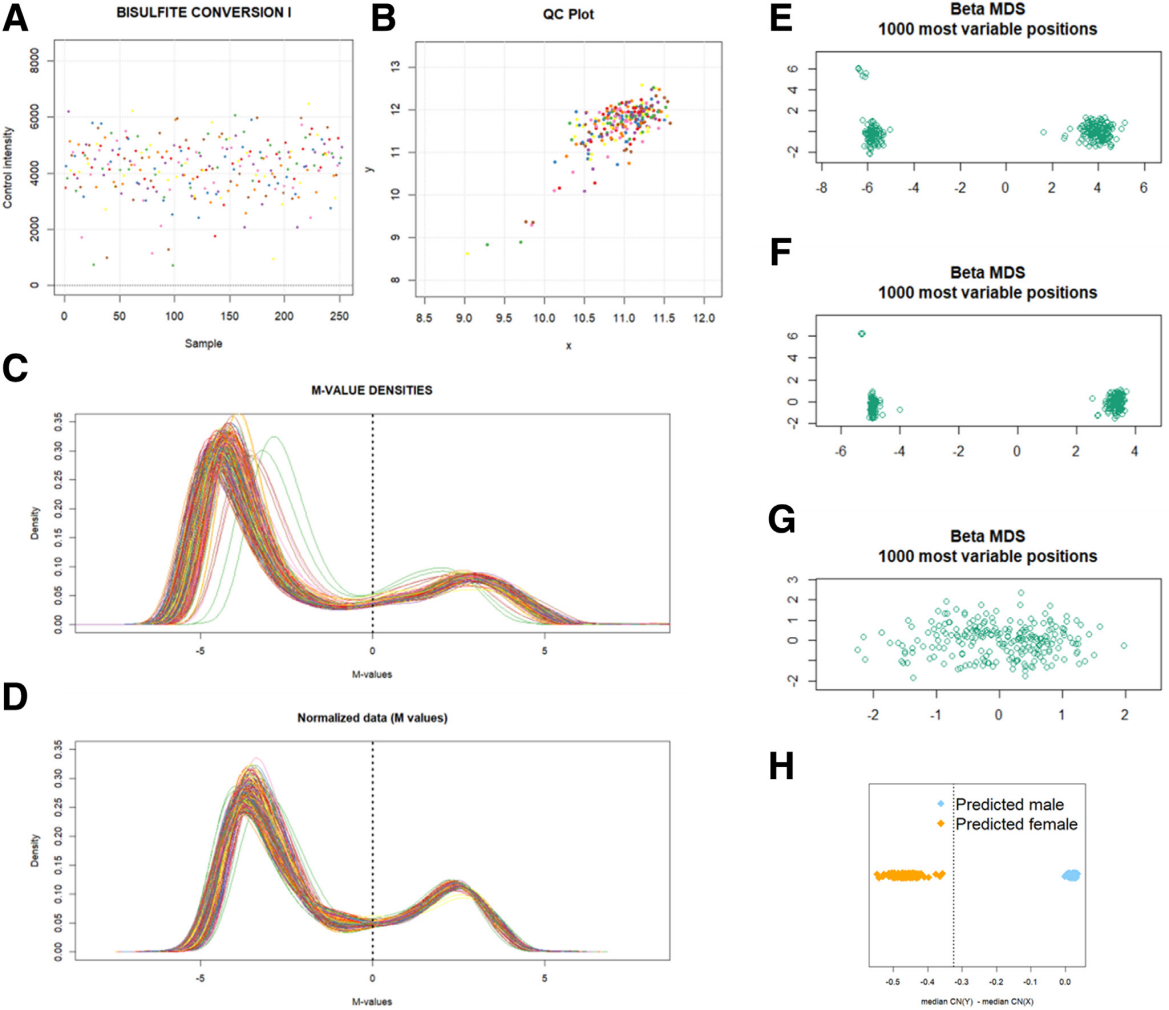
**ShinyMethyl output for quality control for TOPPIC methylation data.** (*A*) Average negative control probe intensities. (*B*) Median intensity of M channel against median intensity of the u channel. (*C, D*) M-value intensities before and after functional normalization. (*E–G*) MDS during processing steps. (*E*) Raw data. (*F*) Following quantile normalization. (*G*) Following filtering of SNPs and sex chromosomes. (*H*) ShinyMethyl sex-prediction plot. No samples were mismatched for sex.

**Figure 3:**
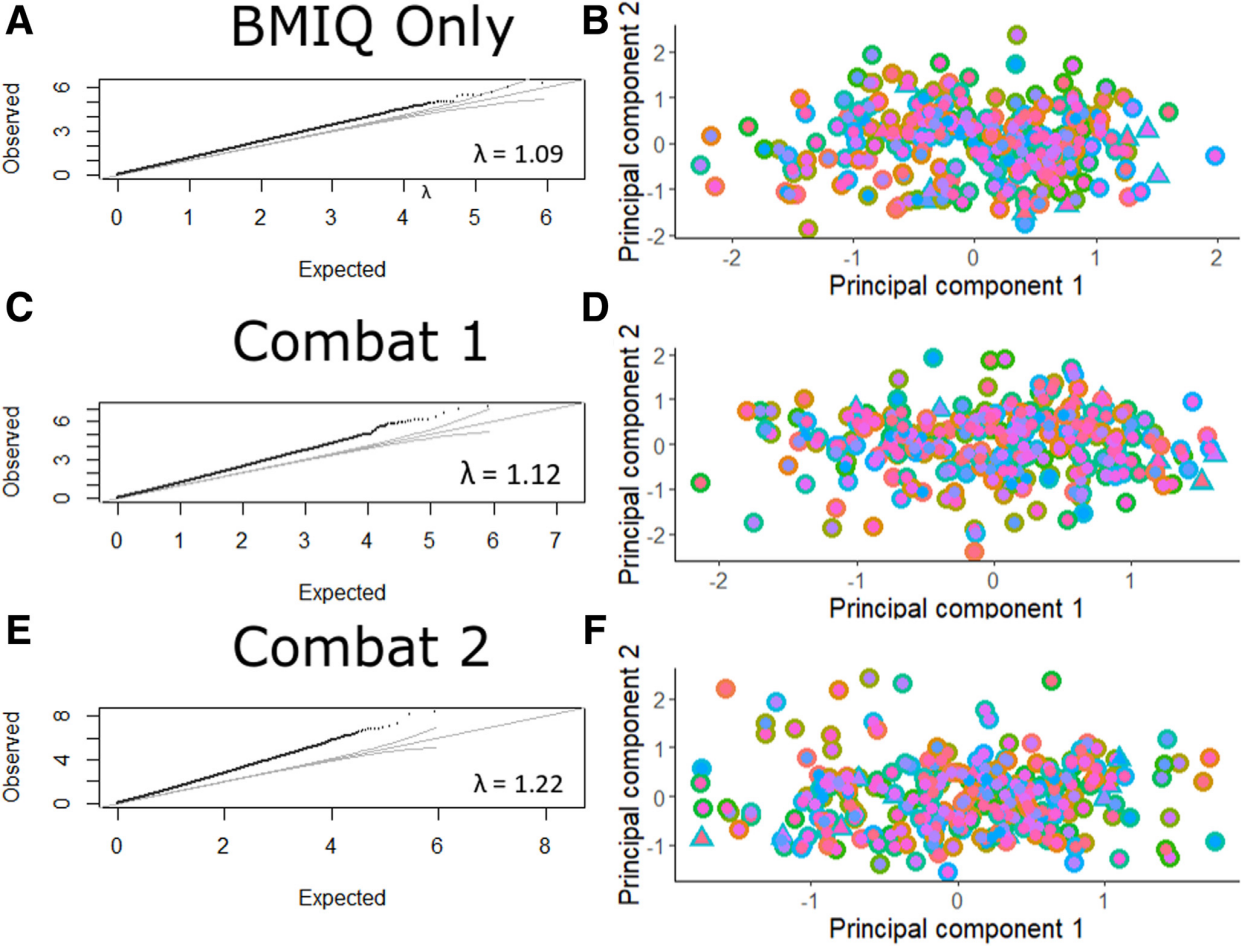
**Batch correction for TOPPIC-only methylation cohort.** (*A, C, E*) QQ plots and Lambda values for the (*A*) TOPPIC cohort following BMIQ and quantile normalization, (*C*) Combat correction for Chip (21 batches), and (*E*) Combat correction for position on array (12 batches). (*B, D, F*) Multidimensional scaling plots showing the first 2 principal components (*B*) TOPPIC cohort following BMIQ and quantile normalization, (*D*) Combat correction for Chip (21 batches), and (*F*) Combat correction for position on array (12 batches). *Inner color*, between array batch; *outer color*, intra-array batch; *triangles*, technical replicates.

**Figure 4:**
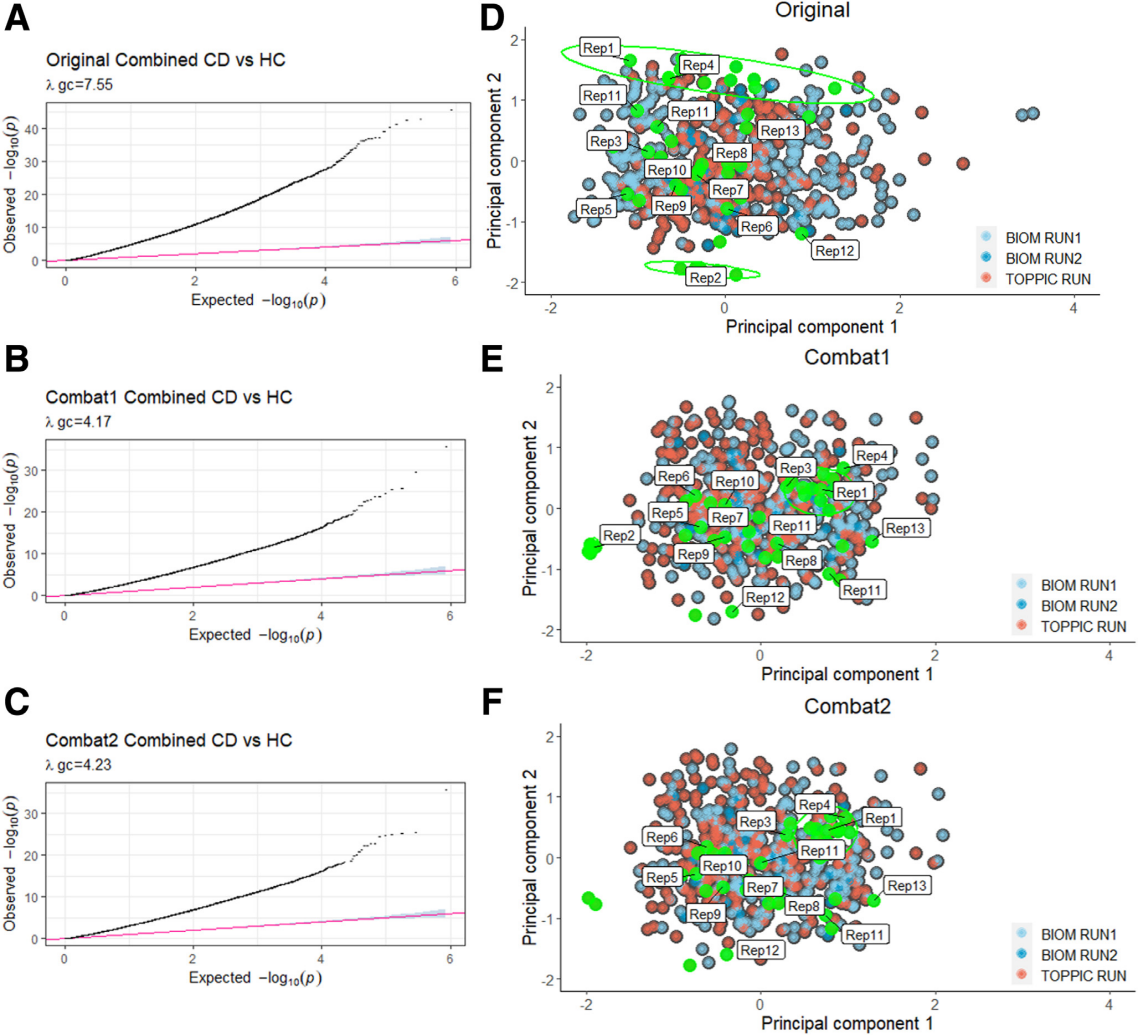
**QQ plots and Lambda values for the originally combined TOPPIC and BIOM datasets following BMIQ and quantile normalization (*A*), followed by Combat correction for methylation chip (*B*) and location within each chip (*C*).** MDS scaling plot of the first 2 principal components for the originally combined of TOPPIC and BIOM datasets following BMIQ and quantile normalization (*D*), followed by Combat correction for methylation chip (*E*), and location within each chip (*F*). Colors (*blue*, *red*) denote different experimental batches. *Green* labelled points denote technical replicates included across chips, plates, runs, and batches.

**Figure 5:**
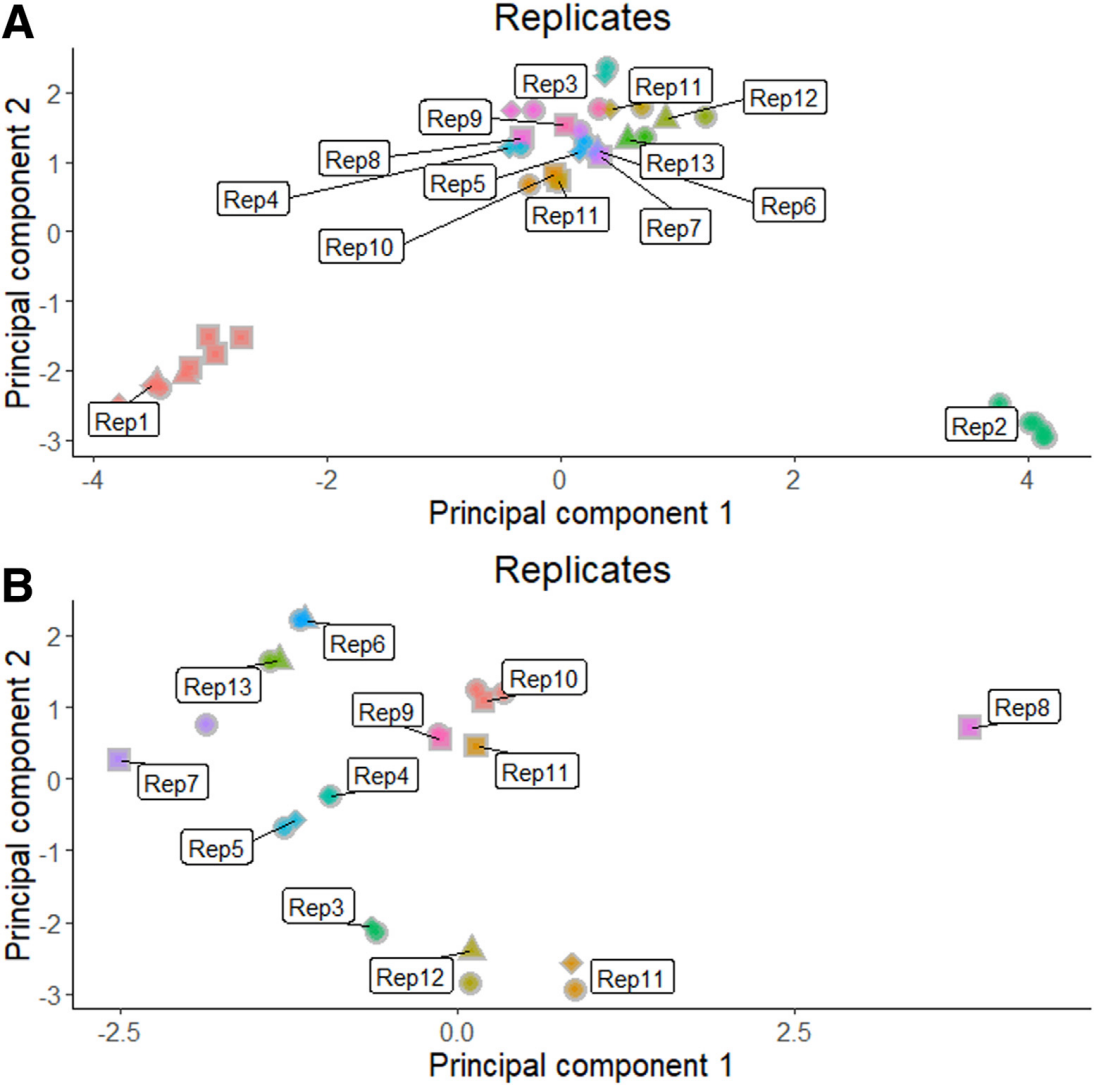
**Principal component plot of the first 2 components (PC1, PC2) using 1000 most variable probes of the combined TOPPIC and BIOM cohorts.***Colors* correspond to technical replicates. *Shapes* refer to 450K scan date. (*A*) All replicates. (*B*) Replicates 1 and 2 removed.

In addition to the 233 novel TOPPIC samples described previously, there were 123 CD samples (combined = 356 CD samples) and 198 control subjects from the IBD-BIOM cohort.^9^ Raw methylation from both cohorts (TOPPIC and BIOM) was normalized together. QQ plots, Lambda values, and clustering of cohorts on MDS plots improved following combat correction (for array number and intra-array position) between TOPPIC and BIOM cohorts (Figure 4). There were more than 40 technical replicates included across experimental batches with good visual clustering on MDS plots (Figure 5). Demographic details from the IBD-BIOM cohort are summarized in Table 2.

**Table 2:** Demographics of IBD-BIOM Validation Set

	CD (n = 123)	SC (n = 84)	*P* value SC vs CD	Healthy volunteers (n = 114)	*P* value HL vs CD
Age, median (IQR)	32.4 (24.9–50.7)	32.8 (26.4–45.5)	.9^a^	32.3 26.4–40.6)	.4^a^
Females, n (*%*)	58 (47.9)	39 (52.7)	.6^b^	59 (50.4)	.8^b^
Smoking status					
Current	53	17	Current vs ex/never	24	Current vs ex/never
Ex	29	17	.005	32	.0005
Never	39	40	Ever vs never	56	Ever vs never
Unknown	0	0	.004	5	.008
CRP	8 (2–23)	0 (0–3.5)	.006^a^		
ESR	18 (5–39)	6 (4.5–7.5)	.002^a^		
FC	495 (135–828)	19 (19–37)	.0001^a^		

CD, Crohn’s disease; CRP, C-reactive protein; ESR, erythrocyte sedimentation rate; FC, fecal calprotectin; HL, healthy laboratory volunteers; IQR, interquartile range; SC, symptomatic control subjects; UC, ulcerative colitis.

Adapted with permission from Ventham et al.

a Wilcoxon rank sum test.

b Chi-square test.

### DNA Methylation and Risk of Disease Recurrence Following Surgery in Patients With CD

#### Differentially Methylated Positions

**Figure 6:**
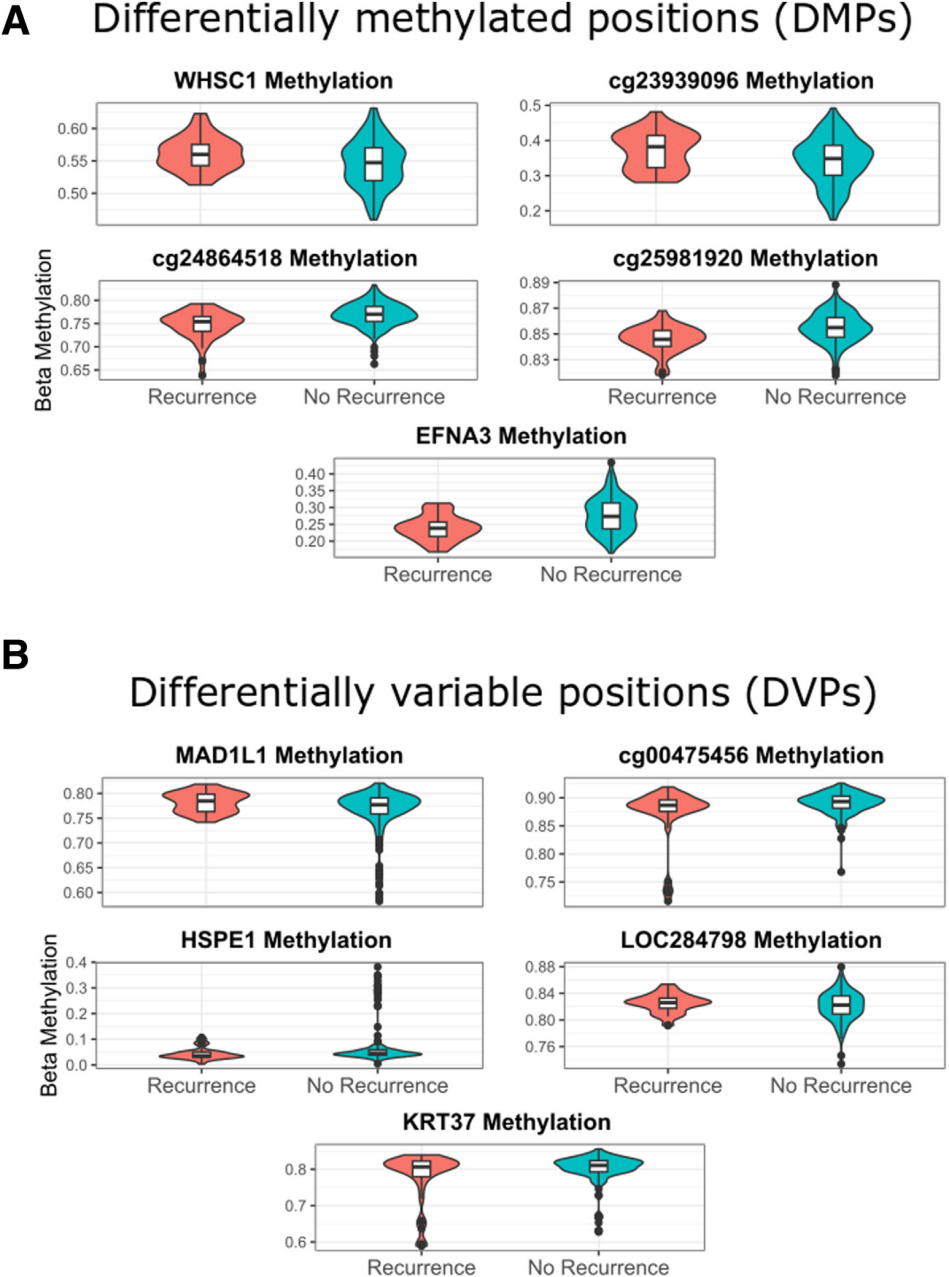
**(*A*) Violin and box plots of DMPs associated with disease recurrence (clinical end point) following surgery for Crohn's disease.** (*B*) Violin and box plots of DVPs associated with disease recurrence (clinical end point) following surgery for Crohn's disease (defined as increase in Crohn’s disease activity index of more than 150 and an increase of 100 points from baseline measurement and institution of immunosuppressive treatment or further surgery).

There were 229 patients within the TOPPIC cohort available for comparison of the primary clinical end point of disease recurrence (n = 42) versus no recurrence. There were 5 statistically significant differentially methylated positions (DMPs) for the primary clinical end point, when including covariates (age, sex, smoking status, placebo/treatment, and estimated cell counts) and adjusting for multiple testing (Figure 6*A*, Table 3). DMPs are cg09916234 (*NSD2/WHSC1*, *P* = 4.07 × 10^-9^, Holm adjusted *P* = .002), cg24864518 (*P* = 7.87 × 10^-9^, Holm adjusted *P* = .003), cg06058618 (*EFNA3*, *P* = 4.92 × 10^-9^, Holm adjusted *P* = .02), cg23939096 (*P* = 1.01 × 10^-7^, Holm adjusted *P* = .04), and cg25981920 (*P* = 1.11 × 10^-7^, Holm adjusted *P* = .048). When smoking is not included as a covariate in the linear model, there were 6 significant DMPs, with cg21472517 (*SAMD1*) in addition to the 5 outlined previously. There were no significant DMPs when comparisons of the endoscopic outcomes were used (data not shown).

**Table 3:** The 5 Holm Corrected Significant Differentially Methylated Positions Associated With Disease Recurrence Versus No Recurrence in Patients Undergoing Surgery for Crohn's Disease

	logFC	sym	Feature	*P* value	Holm adjusted *P* value
cg09916234	0.023	WHSC1	Body	4.07E-09	.002
cg24864518	-0.026	∗	None	7.87E-09	.003
cg06058618	-0.046	EFNA3	Body	4.92E-08	.021
cg23939096	0.017	∗	None	1.01E-07	.043
cg25981920	-0.010	∗	None	1.11E-07	.048

Feature, location of methylation probe in relation to nearby gene on the 450K annotation manifest; logFC, log fold change; sym, gene symbol associated with methylation probe on the 450K annotation manifest.

#### Differential Variable Positions

Differential methylation variability was assessed using the iEVORA method comparing patients with disease recurrence and those without (using the clinical end point). There were 18 differentially variable positions (DVPs) associated with disease recurrence. When covariates were additionally used (age, sex, smoking status, cell proportions), there were 5 significant DVPs associated with disease recurrence (Figure 6*B*, Table 4). The 5 DVPs are cg24696067 (*MAD1L1*, *P* = 6.43 × 10^-6^), cg02208776 (*HSPE1*, *P* = .001), cg18068256 (*KRT37*, *P* = .02), cg00475456 (*P* = .03), and cg20310608 (*LOC284798*, *P* = .03). There were no intersecting DMPs and DVPs associated with disease recurrence.

**Table 4:** Top Table of Differentially Variable Positions in Patients With Disease Recurrence Postresection for CD Using Clinical End Point^a^

	Gene symbol	Chr	Feature	Mean beta no recurrence	Mean beta recurrence	Mean diff	*P* value.t
cg24696067	MAD1L1	7	Body	-0.003	0.015	0.018	6.43E-05
cg02208776	HSPE1	2	1stExon	0.004	-0.018	-0.022	0.001
cg18068256	KRT37	17	Body	0.005	-0.022	-0.026	0.024
cg00475456	∗	1		0.003	-0.014	-0.017	0.027
cg20310608	LOC284798	20	TSS200	-0.001	0.003	0.004	0.031

NOTE. Calculated with the iEVOR method. A matrix of residual methylation values of a linear model including the following covariates was used (age, sex, smoking status, treatment/placebo, cell proportions).

a Defined as increase in Crohn’s disease activity index of more than 150 and an increase of 100 points from baseline measurement and institution of immunosuppressive treatment or further surgery.

The biologic and functional relevance of DMPs and DVPs associated with CD recurrence following surgery are outlined in Table 5.

**Table 5:** Functional and Biologic Relevance of Significant Differential Methylated Positions and Differentially Variable Positions Associated Crohn's Disease Recurrence Following Surgery

Probe	Symbol	Function/relevance in inflammatory bowel disease
Differentially methylated probes		
cg09916234	NSD2/ WHSC1	Nuclear receptor binding SET domain protein 2. Wolf-Hirschhorn syndrome, a multisystem chromosomal disorder associated with a deletion on chromosome 4. Also the probe maps close to the transcription start site of microRNA-943 that has been shown to accelerate airway inflammation in asthma.^19^
cg24864518	∗	This probe maps to an intergenic region close to the TSS of RASGEF1b, a guanine nucleotide exchange factor for Rap2, a member of the family of Rap G-protein signallers.^20^
cg06058618	EFNA3	Ephrine A3. Tyrosine kinase family of receptors. Ephrine-mediated repulsion of cells have a role in maintaining the integrity of the gut epithelial layer and may modulate T-cell activation.^21^ Have previously been implicated in Crohn’s disease^22^ and ulcerative colitis,^23^ and have been postulated as a potential therapeutic target in Crohn’s disease.^24^ Also extensively implicated in gastric and hepatocellular cancers. Target of miR-210-3p.
cg23939096	∗	Maps to a noncoding area.
cg25981920	∗	Maps to a noncoding area close to LY6L lymphocyte antigen6 family member.
Differentially variable probes		
cg24696067	MAD1L1	Mitotic arrest deficient like 1 acts as a spindle assembly checkpoint between metaphase and anaphase. *MAD1L1* was a key finding in our previous work that demonstrated IBD-specific correlation between DNA methylation and gene expression.^10^ A different probe mapping to *MAD1L1* was differentially methylated in colonic intraepithelial cells in UC.^25^ Because of its role in regulating the cell cycle, MAD1L1 is also implicated in a variety of cancers.
cg02208776	HSPE1	Heat shock protein family E that acts a chaperonin. Implicated in colorectal cancer.^26^^,^^27^
cg18068256	KRT37	Keratin 37, a type I keratin that dimerises with type II keratins to form hair and nails.
cg00475456	∗	Maps to an intergenic region close to PLXNA2 a plexin related in axon/nervous system development.
cg20310608	LOC284798	Uncharacterized LOC284798/ENSG00000230725.

IBD, inflammatory bowel disease; UC, ulcerative colitis.

#### Methylated Quantitative Trait Loci

**Figure 9:**
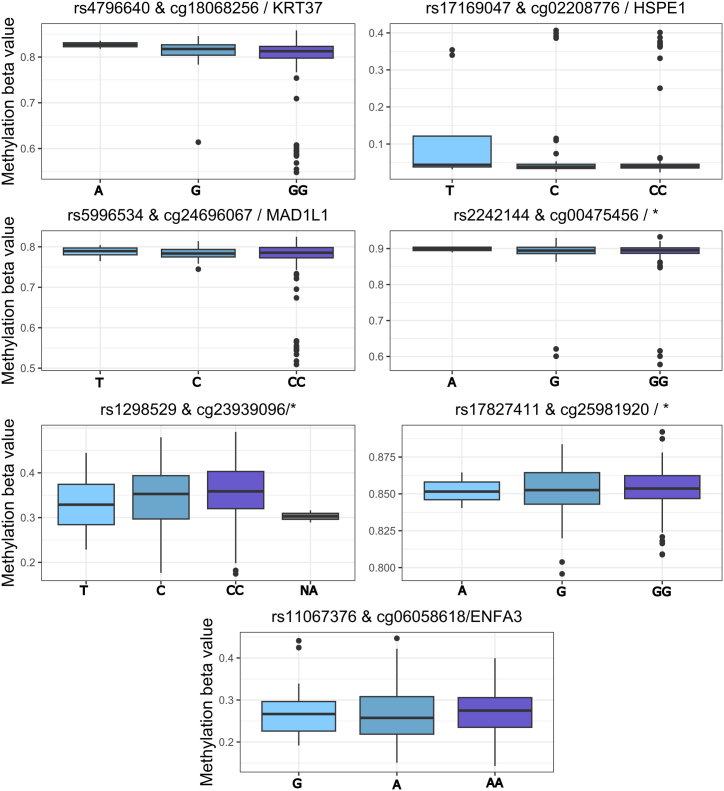
**cis meQTLs of DVP/DMP probes.** Top SNP shown. Age, sex, smoking status used as covariates. MAF of <10% filtered. Cis distance 1 × 10^6^, *P* value threshold <2 × 10^-6^.

**Figure 10:**
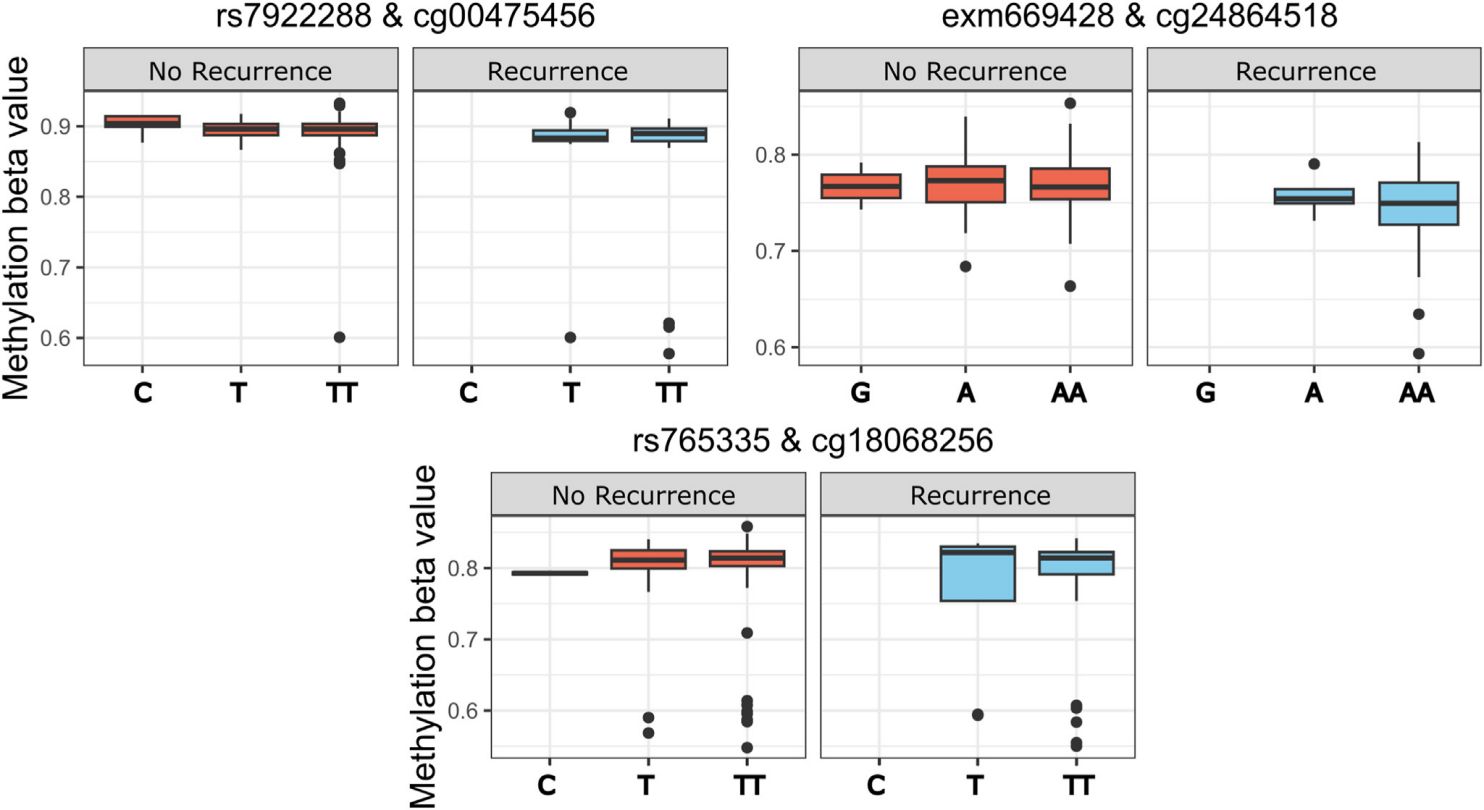
**Cis meQTLs of DVP or DMP probes associated with Crohn’s disease recurrence following surgery.** Top SNP shown. Age, sex, smoking status used as covariates. MAF of <10% filtered. Cis distance 1 × 10^6^, *P* value threshold <2 × 10^-6^.

There were 216 samples with paired methylation and genotype data available for methylated quantitative trait loci (meQTL) analysis. The 5 DMPs and 5 DVP methylation probes were investigated for genotype association (meQTLs) using age, sex, and smoking status as covariates. There were 35 cis meQTLs with a false discovery rate (FDR; *P* < .05), consisting of 35 different single-nucleotide polymorphisms (SNPs) and 7 of the 10 CpGs (Figure 9, Table 6). Three methylation probes had meQTLs associated with the primary end point of CD recurrence (with age, sex, and smoking status as covariates); cg00475456 (∗, DVP, rs7922288, FDR *P* = 4.89 × 10^-20^), cg18068256 (DVP [*KRT37*] rs765335, FDR *P* = 2.80 × 10^-8^), and cg24864518 (DMP, ∗, exm669428, FDR *P* = 1.23 × 10^-5^) (Table 7, Figure 10). Two DMPs (c09916234 [*WHSC1/ NSD2]* and cg2484518) did not show any genetic association.

**Table 6:** Top Cis meQTLs Associated With DMP (black) and DVP (grey) Methylation Probes, Most Significant SNP is Listed (age, sex, smoking status used as covariates)

SNP	Methylation probe/annotation symbol	Statistic	*P* value	FDR corrected *P* value	beta
rs4796640	cg18068256 / KRT37	8.2	1.77E-14	9.33E-08	0.07
rs17169047	cg02208776 / HSPE1	-6.2	2.34E-09	0.003	-0.06
rs5996534	cg24696067 / MAD1L1	5.5	1.32E-07	0.09	0.04
rs2242144	cg00475456 /∗	5.4	1.48E-07	0.09	0.03
rs1298529	cg23939096/∗	5.3	2.50E-07	0.09	0.04
rs17827411	cg25981920 /∗	5.2	3.87E-07	0.12	0.01
rs11067376	cg06058618 / ENFA3	5.0	1.16E-06	0.24	0.03

NOTE. MAF of <10% filtered. Cis distance 1 × 10^6^, *P* value threshold <2 × 10^-6^.

DMP, differentially methylated position; DVP, differentially variable position; FDR, false discovery rate; meQTL, methylated quantitative trait loci; SNP, single-nucleotide polymorphism.

**Table 7:** Top Cis meQTLs Associated With Clinical End Point (Disease Recurrence) DMP (black) and DVP (grey) Methylation Probes, Top SNP is Listed

SNP	Methylation probe	Statistic	*P* value	FDR corrected *P* value	Beta
rs7922288	cg00475456 /∗	12.4	9.27E-27	4.89E-20	0.19
rs765335	cg18068256 / KRT37	7.6	1.30E-12	2.80E-08	0.23
exm669428	cg24864518 /∗	6.2	2.64E-09	1.23E-05	0.10

NOTE. Age, sex, smoking status used as covariates. MAF of <10% filtered. Cis distance 1 × 10^6^, *P* value threshold <2 × 10^-6^.

DMP, differentially methylated position; DVP, differentially variable position; FDR, false discovery rate; meQTL, methylated quantitative trait loci; SNP, single-nucleotide polymorphism.

### Validation of DNA Methylation Changes in IBD Cases and Control Subjects

#### TOPPIC CD Versus BIOM Control Subjects

There were 19,179 DMPs (Holm adjusted *P* < .05) associated with CD (TOPPIC, n = 233) compared with BIOM control subjects (n = 198, in Table 8). Significant DMPs include our previously identified CD-associated DMPs including *RPS6KA2* (Holm adjusted *P* = 1.2 × 10^-19^) and *SBNO2 (*Holm adjusted *P* = 1.2 × 10^-11^). Of the 412 CD-specific DMPs identified originally in Ventham et al,^9^ 80 overlapped with the TOPPIC alone dataset (19.4%, with good correlation of log fold change values; Pearson correlation = 0.97). Using DMRCate, there were 4099 CD-associated differential methylated region (DMRs) with an FDR *P* < .00001. This included our previously described DMRs (*TXK* [FDR *P* = 3.6 × 10^14^], *WRAP73* [FDR *P* = 1.9 × 10^9^], *ITGB2* [FDR *P* = 1.4 × 10^7^], and *VMP1* [FDR *P* = 1.7 × 10^7^]).

**Table 8:** Top 20 Differentially Methylated Positions Crohn's Disease TOPPIC (n = 233) Versus Control Subjects BIOM Dataset (n = 198)

	logFC	sym	Feature	*P* value	Adjusted *P* value
cg21155778	-0.10	FGD6	TSS200	8.65E-50	3.72E-44
cg17931986	0.07	COL11A2	3'UTR	2.23E-38	9.60E-33
cg19755108	0.03	UIMC1	TSS1500	1.96E-37	8.44E-32
cg19056176	-0.06	KIAA0513	5'UTR	1.21E-36	5.21E-31
cg14863978	-0.04	GBX1	TSS200	1.30E-36	5.58E-31
cg02159402	-0.09	GALNT11	TSS200	4.71E-36	2.03E-30
cg05410609	-0.07	CCDC85C	5'UTR	5.89E-35	2.53E-29
cg15281724	0.06	TXLNB	Body	1.94E-34	8.32E-29
cg19165344	0.03	AP1B1	TSS1500	2.82E-33	1.21E-27
cg04450857	-0.06	EMX2OS	Body	4.82E-33	2.07E-27
cg17541922	-0.04	PRSS23	5'UTR	7.18E-33	3.09E-27
cg18100079	0.04	∗	None	1.49E-32	6.41E-27
cg02951344	-0.05	SYTL2	TSS200	3.66E-32	1.57E-26
cg14910854	0.03	LOC150776	Body	8.88E-32	3.82E-26
cg11338426	-0.05	CRHR1	1stExon	1.50E-31	6.45E-26
cg03047400	-0.06	∗	None	3.04E-31	1.31E-25
cg18301538	0.04	GBF1	Body	3.47E-31	1.49E-25
cg21120539	-0.04	CTSZ	1stExon	3.79E-31	1.63E-25
cg06996129	-0.09	∗	None	5.89E-31	2.53E-25
cg12216772	-0.07	ANUBL1	5'UTR	1.02E-30	4.39E-25

NOTE. Age, sex, smoking, estimate cell proportion included as covariates in linear model. Holm adjusted *P* value.

logFC, log fold change.

#### Combined Analysis of TOPPIC CD and BIOM CD Versus Control Subjects

There were 4505 DMPs (Holm adjusted *P* < .05) associated with CD (n = 356) compared with control subjects (n = 198, top 20 DMPs presented in Table 9). The top DMP is *RPS6KA2* (Holm adjusted *P* = 1.4 × 10^-29^), the principal finding from our previous work, and the top 20 includes 2 probes within *SBNO2* (Holm adjusted *P* = 1.9 × 10^-18^). There was 86.8% overlap (3909) of DMPs those identified using TOPPIC samples alone and the combined analysis with strong correlation of log fold change (Pearson correlation = 0.99). Using DMRCate, there were 812 CD-associated DMRs with an FDR *P* < .00001. This included our previously described DMRs (*TXK* [FDR *P* = 4.4 × 10^-12^], *VMP1* [FDR *P* = 6.3 × 10^11^], *WRAP73* [FDR *P* = 5.3 × 10^8^], *ITGB2* [FDR *P* = 5.2 × 10^7^]).

**Table 9:** Combined-Analysis of Differentially Methylated Positions Crohn’s Disease (TOPPIC and BIOM) Versus Control Subjects BIOM Dataset)

	logFC	sym	Feature	*P* value	Adjusted *P* value
cg17501210	-0.06	RPS6KA2	Body	3.15E-35	1.35E-29
cg21155778	-0.06	FGD6	TSS200	4.10E-26	1.76E-20
cg25422678	0.03	BRE	Body	3.06E-25	1.32E-19
cg24430034	0.03	∗	None	4.15E-25	1.78E-19
cg18181703	-0.04	SOCS3	Body	1.86E-24	8.00E-19
cg18608055	-0.05	SBNO2	Body	4.39E-24	1.89E-18
cg03546163	-0.07	FKBP5	5'UTR	4.39E-22	1.89E-16
cg26955383	0.03	CALHM1	TSS200	4.90E-22	2.10E-16
cg26470501	-0.03	BCL3	Body	5.16E-22	2.22E-16
cg07573872	-0.05	SBNO2	Body	2.25E-21	9.66E-16
cg16411857	-0.04	NLRC5	TSS1500	2.61E-21	1.12E-15
cg12992827	-0.05	∗	None	1.13E-20	4.84E-15
cg04975846	-0.05	TRAPPC2L	Body	5.67E-20	2.44E-14
cg07839457	-0.07	NLRC5	TSS1500	5.81E-20	2.50E-14
cg12269535	-0.04	SRF	Body	6.29E-20	2.71E-14
cg09090048	-0.04	VPS26B	TSS1500	7.91E-20	3.40E-14
cg11738543	-0.02	SOCS2	Body	2.54E-19	1.09E-13
cg02508743	0.03	LYN	Body	3.14E-19	1.35E-13
cg08791347	0.03	FRMD4A	Body	1.12E-18	4.83E-13
cg01839860	0.02	UBE2D2	5'UTR	1.99E-18	8.55E-13

NOTE. Age, sex, smoking, estimate cell proportion included as covariates in linear model. Holm adjustment for multiple testing.

logFC, log fold change.

#### Differentially Variable Positions (CD vs BIOM Control Subjects)

Differential variability was performed comparing CD cases (BIOM CD and TOPPIC) versus control subjects (BIOM control subjects) using the iEVORA method.^28^ There were 18,993 DVPs hypervariable in CD compared with BIOM control subjects. Previously described IBD-associated DMPs were included as DVPs (*SBNO2*, var log 2 = 0.8, 1.2 × 10^-32^; *RPS6KA2*, var log 2 = 0.8, *P* = 5.5 × 10^-11^ [uncorrected *t*-test]).

### Smoking and Epigenetic Age

#### Smoking

**Figure 7:**
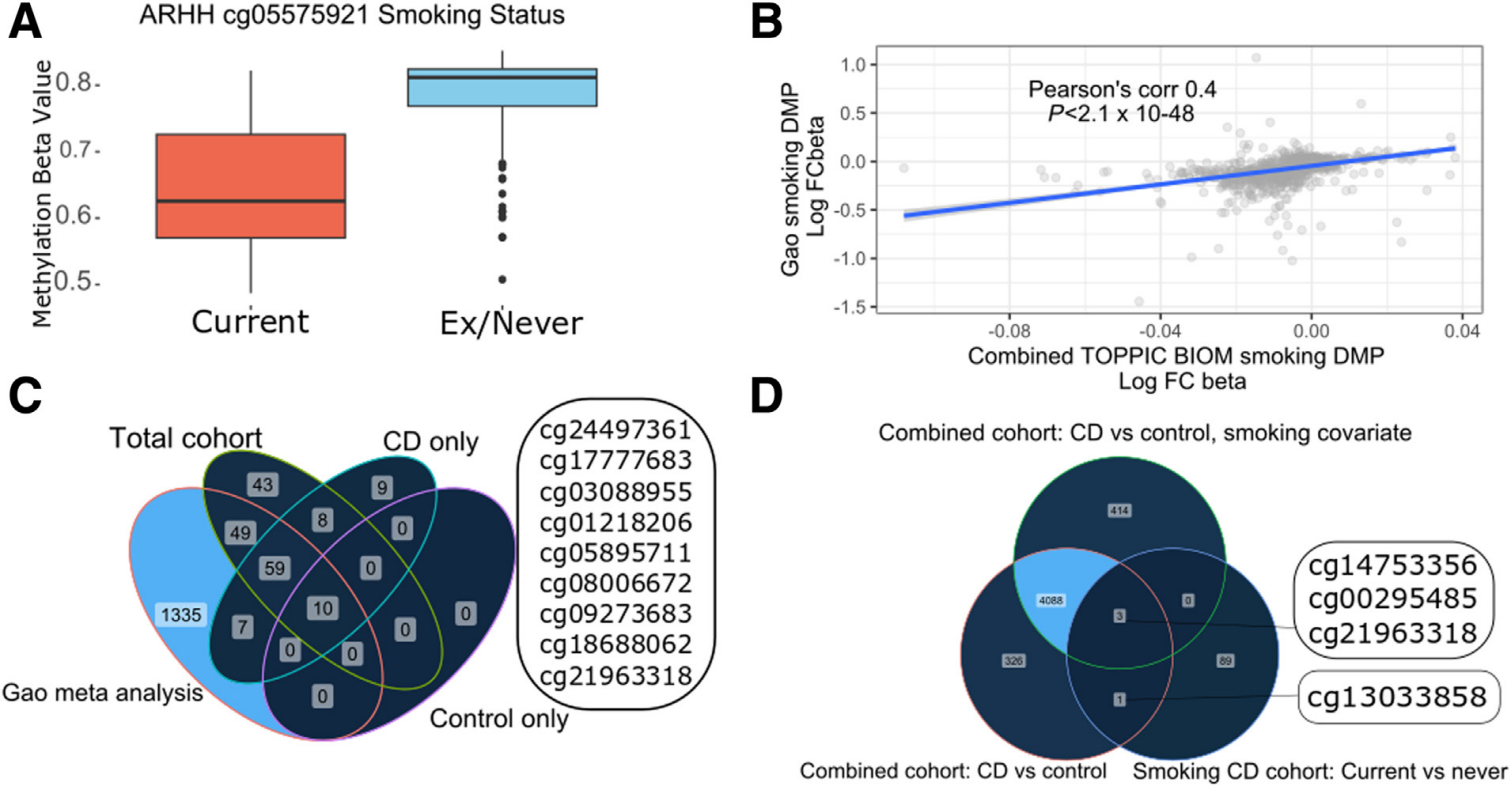
**(*A*) Aryl hydrocarbon receptor repressor ARHH/cg05575921 methylation in smokers and nonsmokers and exsmokers in the entire cohort (combined CD and control subjects in both cohorts).** (*B*) Correlation plot between smoking and exsmoker/nonsmoker log fold change beta value in Gao et al meta-analysis and in the present study (DMPs, Holm *P* < .05, entire cohort CD and control subjects combined). (*C*) Venn diagram of overlapping probes in Gao et al meta-analysis, smokers versus nonsmokers (DMPs, Holm *P* < .05) in total cohort (CD and control subjects combined), Crohn’s disease patients only, and control subjects only. The 9 smoking-associated CpGs seen only in the Crohn’s cohort are listed in the box. (*D*) Venn diagram of Crohn’s disease versus control DMPs in the entire cohort without using smoking as a covariate, entire cohort using smoking as a covariate, and in the smoking-associated DMPs in the Crohn’s only cohort. There are 4 CpGs that overlap that are both associated with Crohn’s (vs control, DMPs) and smoking (smoking vs exsmoker/never smoker, DMPs) that are listed in the boxes.

We performed a methylation analysis of smokers versus exsmokers and nonsmokers using the combined cohort (n = 554, regardless of case or control status). There were 169 methylation probes that associated with smoking (Holm corrected <0.05). Aryl hydrocarbon receptor repressor (AHRR) methylation has been strongly associated with smoking status and we confirm hypomethylation in current smokers (cg05575921, beta difference -10.8, Holm adjusted *P* = 5.46 × 10^-45^; Figure 7*A*) with 5 *AHRR* probes in the top 20 most significant probes (cg05575921, cg21161138, cg26703534, cg14817490, cg25648203; Table 10).

**Table 10:** Top 20 Smoking-Associated Methylation Probes

	logFC	*P* value	Adjusted *P* value	SYM	Feature
cg05575921	-0.108	1.27E-50	5.46E-45	AHRR	Body
cg01940273	-0.062	1.95E-41	8.38E-36	∗	None
cg03636183	-0.072	1.38E-35	5.92E-30	F2RL3	Body
cg06126421	-0.071	1.24E-32	5.33E-27	∗	None
cg05951221	-0.068	8.27E-31	3.56E-25	∗	None
cg21161138	-0.040	6.87E-29	2.95E-23	AHRR	Body
cg26703534	-0.035	1.84E-28	7.89E-23	AHRR	Body
cg21566642	-0.056	2.24E-23	9.61E-18	∗	None
cg03329539	-0.031	4.73E-22	2.03E-16	∗	None
cg14817490	-0.043	2.91E-20	1.25E-14	AHRR	Body
cg25648203	-0.029	6.33E-20	2.72E-14	AHRR	Body
cg04885881	-0.040	7.50E-20	3.23E-14	∗	None
cg09935388	-0.063	9.17E-19	3.94E-13	GFI1	Body
cg19572487	-0.033	3.40E-18	1.46E-12	RARA	5'UTR
cg07826859	-0.026	4.95E-17	2.13E-11	MYO1G	TSS1500
cg14753356	-0.035	5.37E-17	2.31E-11	∗	None
cg25189904	-0.054	9.99E-17	4.30E-11	GNG12	TSS1500
cg07339236	-0.028	2.45E-16	1.05E-10	ATP9A	Body
cg00310412	-0.027	2.85E-16	1.22E-10	SEMA7A	Body
cg23079012	-0.024	7.46E-16	3.21E-10	∗	None

NOTE. Linear model smokers versus exsmokers and nonsmokers including age, sex, and cell admixture as covariates. Data include all patients and control subjects in combined cohort (CD and control subjects). Adjusted *P* value is Holm correction for multiple testing.

logFC, log fold change.

Of the 169 significant probes, 137 (81%) have previously been described by Gao et al^29^ in a meta-analysis of smoking-related probes. There was a modest but significant correlation in log fold difference in beta values here and published by Gao et al^29^ (Pearson R = 0.4; Figure 7*B*).

To delineate CD-specific smoking associated methylation we then analyzed smoking-related methylation in CD cases (n = 356) and control subjects (n = 198) separately. There were 9 CpGs associated with smoking in patients with CD that did not overlap with the control or combined cohort or CpGs that had previously been described by Gao et al^29^ (cg24497361, cg17777683, cg03088955, cg01218206, cg05895711, cg08006672, cg09273683, cg18688062, cg21963318; Figure 7*C*). When comparing these CD-specific smoking-associated CpGs, there were 4 overlapping probes compared with the CD case control DMPs described in the replication analyses later (cg14753356, cg00295485, cg21963318, cg130338858; Figure 7*D*). The functional relevance of these smoking-related methylation probes is detailed in Table 11.

**Table 11:** Functional and Biologic Relevance of Smoking-Related Probes Associated With CD

Probe	Symbol	Function/relevance in IBD
CD-specific smoking-associated methylation probes		
cg24497361	RHOG	Ras homologue family member G. Rho family of small GTPases. Facilitates translocation of a GEF from the cytoplasm to the membrane. Found to be a smoking-related methylation probe by Dugue et al.^30^
cg17777683	CFLAR (cFLIP)	Caspase 8 and FADD-like apoptosis regulator. Regulator of apoptosis. Cigarette smoke decreases bronchial expression and increases susceptibility for cell death and DAMP release.^31^ Found to be smoking-related by Sikdar et al.^32^
cg03088955 Table 10 - Top 20 Smoking associated methylation probes.	JOSD1	Josephin containing domain 1. Deubiquitinization enzyme. Involved in autophagy.^33^
cg01218206	SIK2	Salt-induced kinase 2. Enable ATP binding activity. Involved in positive regulation of TORC1 and 2 signaling. Involved in TGF-β mediated apoptosis.^34^ Found to be a smoking-related methylation probe by Dugue et al.^30^
cg05895711		Found to be a smoking-related methylation probe by Dugue et al.^30^
cg08006672		Seen by Sikdar et al^32^ in a meta-analysis of smoking-related probes to associate with smoking.
cg09273683	PIP4KA2	Phosphatidylinositol-5,4-biphosphate 4 kinase type alpha 2. Kinase involved in secretion, cell proliferation, differentiation, and motility. Linked with schizophrenia and acute myeloid leukemia. A SNP in this region was found to be an environmental interactor between smoking and colorectal cancer.^35^
cg18688062	PSORS1C3	Psoriasis Susceptibility 1 Candidate 3. Found to be a smoking-related methylation probe by Dugue et al.^30^
cg21963318	COX4I1	Cytochrome c oxidase, a mitochondrial enzyme involved in mitochondrial respiration.
Smoking-related probe that intersects with CD vs control DMPs (smoking included as a covariate)		
cg14753356		Found to be smoking related by Sikdar et al^32^ and Dugue et al.^30^
cg00295485	UXS1	Found to be smoking related by Sikdar^32^
cg21963318	COX4I1	Cytochrome c oxidase, as above
Smoking-related probe that intersects with CD vs control DMPs (smoking not a covariate)		
cg13033858	SSH1	Slingshot protein phosphatase 1. Associated with colorectal cancer progression and prognosis.^36^ Found to be smoking related by Sikdar et al.^32^

NOTE. Yellow denotes probes not associated with smoking in previous meta-analyses of smoking and methylation (Gao, Sikar, Dugue et al).

CD, Crohn’s disease; DAMP, damage associated mucosal patterns; DMP, differentially methylated position; GEF, guanine nucleotide exchange factor; IBD, inflammatory bowel disease; SNP, single-nucleotide polymorphism.

#### Epigenetic Age

**Figure 8:**
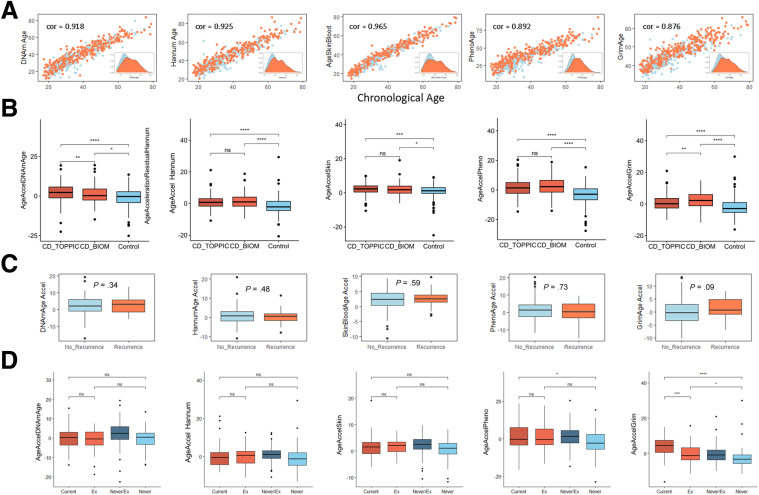
**Epigenetic age analysis using methods by (from right to left) Horvath (DNAmAge),**^40^**Hannum,**^41^**tissue specific (skin and blood clock),**^21^**phenoAge,**^42^**and GRIMage clocks.**^22^ (*A*) Correlation plot of methylation age (y-axis) and biologic age (x-axis) using methods above, inset, density plot of methylation age). Cor, Pearsons R Correlation estimate. (*B*) Boxplots of age acceleration using methods above in patients with Crohn’s disease requiring surgery (CD_TOPPIC), newly diagnosed Crohn’s disease patients (CD_BIOM), and control subjects. (*C*) Boxplots of age acceleration in patients included in the TOPPIC trial who went on to develop recurrence or no recurrence following surgery. (*C*) Box plot for each methylation clock age acceleration and smoking status, current, exsmoking (recorded in the BIOM cohort), exsmoker/never smoker (grouped together as part of the TOPPIC cohort), and never smoked (recorded in the BIOM cohort). Ns = *P* > .05, ∗*P* < .05, ∗∗*P* < .01, ∗∗∗*P* < .001, ∗∗∗∗*P* < .0001 (Wilcox test).

Methylation age was calculated using the following methods: Horvath (DNAmAge),^37^ Hannum,^38^ phenoAge,^39^ tissue specific (skin and blood clock),^40^ and GRIMage clocks.^41^ All clocks demonstrated a strong and highly significant correlation with the biologic age, with the skin and blood clock demonstrating the strongest correlation (Pearson R = .96; 95% CI, 0.959–0.97; *P* <1 × 10^16^) (Figure 8*A*).

Epigenetic age acceleration is demonstrated in patients with CD compared with control subjects using all clocks (Figure 8*B*). When comparing age acceleration newly diagnosed patients with CD in the BIOM cohort with those with established disease requiring surgery in the TOPPIC cohort, there was some evidence of age acceleration in those requiring surgery using the DNAmAge clock, deceleration using the GrimAge clock, and no difference when using the other 3 clocks. The GrimAge clock also demonstrated some evidence of age acceleration in patients with disease recurrence following surgery compared with those without recurrence (+1.04 years; 95% CI, -0.04 to 2.22; *P* = .09; Figure 8*C*). GrimAge acceleration strongly associated with smoking status (Figure 8*D*), but not inflammatory markers (C-reactive protein: *r* = 0.03, *P* = .6; albumin: *r* = 0.08, *P* = .2).

## Discussion

This study presents a detailed DNA methylation from a multicenter UK randomized controlled trial. We demonstrate differential methylation and differentially variable methylation in patients developing CD recurrence following surgery. Furthermore, the results strongly validate our previous studies,^7^, ^8^, ^9^, ^10^ describing methylation differences in IBD cases versus control subjects, which had involved newly diagnosed patients, rather than those with established disease.

### Prediction of CD Recurrence Following Surgery

#### DMPs

The present study includes a unique and homogenous cohort of patients with CD sampled before surgical resection and followed up within the rigorous confines of a randomized controlled trial with accurate clinical and endoscopic follow-up data. A smaller study with a similar cohort of patients postresection for ileal CD did not demonstrate systemic differences in DNA methylation in those experiencing a recurrence.^42^ We demonstrate 5 significant DMPs following stringent correction for multiple testing. The significant DMPs include *EFNA3*, a tyrosine kinase receptor that plays a role in maintaining gut epithelial integrity and T-cell activation^21^ and has been implicated in CD^28^ and ulcerative colitis.^23^ The ephrines have been postulated as potential therapeutic targets in CD.^24^ *WHSC1/NSD2* is a nuclear binding domain associated with the condition Wolf-Hirschhorn syndrome. Notably the methylation probe exists close to a proinflammatory microRNA (mir-943).^19^

#### DVPs

Most epigenome-wide association studies have focused on case-control quantitative differences in DNA methylation at specific sites (DMPs). In the context of complex diseases such as IBD, the absolute differences in mean DNA methylation are often small (<5%), with unclear biologic consequence. There has been interest in measuring DNA methylation variability, or the pattern of variance at these sites. DVPs have been described as heterogeneous outlier events and first described in cancer but increasingly described in complex diseases including T1 diabetes mellitus and rheumatoid arthritis twin studies.^43^^,^^44^ We have identified 5 DVPs associated with disease recurrence following surgery. The most interesting DVP is *MAD1L1*, a mitotic arrest deficient 1 that represents a spindle assembly checkpoint between anaphase and metaphase. *MAD1L1* was a key finding in our previous work as a DMP that demonstrates IBD-specific appropriate inverse correlation between methylation and gene expression.^10^ *MAD1L1* differential methylation has additionally been seen at the gut level, within intraepithelial cells in ulcerative colitis.^25^ The biologic significance of differentially variability of methylation has not been well delineated. Unlike DMPs, DVPs lack clinical utility biomarkers because this technique relates to groups rather than individual patients.

#### meQTLs

Our group and others have previously demonstrated that genetic variation between IBD cases and control subjects relate to differential methylation,^9^^,^^10^^,^^13^ raising the possibility that methylation may be a mediator of genetic susceptibility. Key DMRs including *VMP1* and *ITGB2* have been shown to be meQTLs.^8^^,^^10^ In the present study, there was a cis-genetic association in 8 of 10 methylation sites of interest (5 DMPs and 5 DVPs). Three meQTLs were associated with disease outcome (cg00475456, cg18068256, cg24864518; Figure 10); however, it is likely that differences are driven by small differences in allele frequency in patients with or without disease recurrence.

#### Smoking

There is a very strong relationship between smoking and CD susceptibility,^45^ behavior,^46^ and with postsurgical recurrence;^47^ indeed in the TOPPIC trial, smoking habit was not only a determinant of recurrence; but also was unexpectedly associated with the efficacy of thiopurine therapy.^18^ The mechanism is uncertain, but given the significant effects of smoking on DNA methylation,^29^^,^^30^^,^^32^ the relationship between smoking, CD, CD recurrence after surgery, and DNA methylation is of particular interest. Using the entire cohort (CD and control subjects), we were able to replicate the previously published smoking-related methylation probes^29^^,^^30^^,^^32^ and correlate beta fold differences between smokers and nonsmokers in ours and published series.^38^ AHRR methylation has been strongly associated with smoking status and we confirm hypomethylation in current smokers (beta difference -10.8; Holm adjusted *P* = 5.46 × 10^-45^) with 5 *AHRR* probes in the top 20 most significant probes. We then looked to identify smoking-associated probes that were present in patients with CD (and not control subjects). There were 3 CD-specific smoking-related probes that had not been associated with smoking in other published series. One probe mapped to *JOSD1* (cg03088955), a disubiquination enzyme with a role in autophagy,^33^ and another mapped to *PIP4KA2* (cg09273683), a gene with an SNP that was found to be an environmental interactor between smoking and colorectal cancer.^35^

#### Epigenetic Clock

DNA methylation data can be used to predict the biologic age of patients/samples and DNAm age acceleration is associated with mortality and a poorer prognosis in a range of conditions.^48^^,^^49^ In the present dataset, we have used an online tool (Clock foundation) to calculate epigenetic age using a range of more recently developed methylation clocks. We observe DNAm age acceleration in patients with CD compared with control subjects, replicating the same finding in our previous work.^10^ Using the GrimAge clock we also demonstrate some evidence of epigenetic age acceleration in patients with CD recurrence following surgery, a finding not observed when using the other clocks. GrimAge may outperform the other clocks when predicting all-cause mortality and other age-related morbidity (healthspan).^50^ The GrimAge clock was developed to include DNAm-based surrogate markers for smoking and other plasma proteins.^41^ Epigenetic age acceleration occurs following major surgery, in particular following emergency hip fracture surgery, but returns to baseline 4–7 days following surgery.^51^^,^^52^ Of more relevance, elective colorectal surgery was not associated with epigenetic age acceleration.^51^^,^^52^ GrimAge acceleration associating with smoking and CD recurrence, but not traditional markers of inflammation, is particularly interesting given that smoking was found to be an important factor for disease recurrence in the original TOPPIC study.

### Replication of CD Versus Control Subjects (Case vs Control)

A significant strength of this large DNA methylation dataset was the ability to validate our previous findings of differential methylation occurring in IBD cases and control subjects.^9^ Critically, this demonstrates validation in a distinct cohort of patients recruited across multiple sites across the United Kingdom. Whereas our previously published case-control analyses involved newly diagnosed patients,^9^ the TOPPIC cohort consists of patients with established disease. Using TOPPIC data we replicated our previous key DMPs *TXK* (FDR *P* = 3.6 × 10^14^), *WRAP73* (FDR *P* = 1.9 × 10^9^), *VMP1* (FDR *P* = 1.7 × 10^7^), and *ITGB2* (FDR *P* = 1.4 × 10^7^). Data from the RISK cohort, a treatment-naive pediatric inception cohort, demonstrated a tendency for most methylation signals to revert following treatment,^13^ notably with the exception of IBD-associated *RPS6KA2* hypomethylation, a finding replicated in using this novel cohort (Holm adjusted *P* = 1.2 × 10^-19^). Data from this present study suggest either that these methylation findings may either endure from diagnosis or, alternatively, be present; resolve in remission; and recur in patients with uncontrolled disease reflecting active inflammation at time of sampling. Although the present study cannot address these issues, longitudinal analysis suggest that for most the loci, resolution may occur with disease control; in a small proportion, including notably *RPSKA2*, the changes may be constant regardless of inflammatory status.^13^ This area is under further analysis.

### Differential Variable Positions

In this study, we describe CD-associated differentially variable methylation for the first time in IBD versus control subjects. The enrichment of DMPs and DMRs is an artefact of the analytical technique, with the iEVORA method ranking DVPs higher if a DMP at genome-wide significance level or as close to possible to a DMP.^28^ Variable methylation has been hypothesized to account for differences in disease susceptibility among individuals and between ethinicites.^53^ It has been noted in healthy individuals that there is higher variability in specific regions of genome, and in particular in immune-related pathways, and low variability in highly conserved regions associated with basic cellular functions.^54^ The pathobiologic significance of the DVPs described here warrants further investigation.

### Strengths and Limitations

This is a large dataset of phenotypically homogenous patients with IBD with established disease and provides complementary information to our previously published work in newly diagnosed patients. The combined datasets provide one of the largest series genome-wide DNA methylation data in CD to date and provides compelling replication of our previous key findings in a novel dataset of patients with established disease. The TOPPIC trial was a well-conducted randomized controlled trial performed across multiple sites across the United Kingdom with well-phenotyped data and accurate follow-up data to 3 years. Raw data were normalized together and included more than 40 technical replicate samples performed across chip positions and across separate methylation runs for each separate cohort (TOPPIC, BIOM), with appropriate clustering on MDS plots increasing the confidence of performing analyses across cohorts (Figure 5), limiting the impact of the control samples arising from 1 of the 2 datasets. Notwithstanding this, novel DMPs described in the TOPPIC CD versus BIOM control subjects require further replication. Despite rigorous correction and technical replicates, results from this analysis are likely to be overinflated, as noted by the number of positive DMPs in the TOPPIC CD versus control subjects being higher than in the combined analysis. The blood sample used for methylation analysis was taken before administration of the study treatment (6-MP) or placebo and will not affect the methylation data itself but may impact the studied outcome of disease recurrence (despite nonstatistically significant findings in original randomized controlled trial). RNA was not available to attempt to associate differential methylation variance and expression.

## Conclusions

We identify methylation changes present at the time of surgery that are associated with future CD recurrence within 3 years. Probes within the 5 site-specific (DMPs) and 5 DVPs associate with the underlying genotype and relate to genes with biologic relevance to CD. Given the relationship between smoking, methylation, and IBD, we have identified CD-specific smoking-related methylation sites. Replication of the CD-associated methylation alterations is achieved, having previously characterized only in adult and pediatric inception cohorts, in patients with well-established disease requiring surgery.

## Methods

### Datasets

TOPPIC was a placebo-controlled, randomized controlled trial of 6-MP at 29 UK centers in patients with CD undergoing ileocolic resection between 2008 and 2012.^18^ Genomic DNA was extracted from whole blood samples from 229 of the 240 patients taken before intestinal surgery. The IBD-BIOM cohort consists of 123 patients with newly diagnosed CD and 198 control subjects, further details of which are described in the original paper (Figure 1).^9^

### Samples

Peripheral blood leukocyte DNA was bisulphite converted and DNA methylation profiling was performed using the Illumina HumanMethylation450K platform (Illumina, San Diego, CA). Samples from patients treated with 6-MP or placebo were randomly distributed across chips. A total 41 technical replicates were distributed across chips, runs, and cohorts. Genotype analysis was performed using the Illumina Omni Express Exome (500k SNPs) array for the TOPPIC cohort and the Illumina CoreExome Beadchip array.

### DNA Methylation Analysis

#### Data Preprocessing

DNA methylation data was read from iDats using the R package minfi.^55^ Estimated cell proportion admixture^56^ was obtained using estimateCellCounts function of the same package. The minfi processing stream was then followed: quantile normalization (preprocessQuantile); probes on sex chromosomes were removed (11458 probes), samples with >1% with detection *P* values >5% (0 samples) were filtered; and methylation probes containing SNPs (dropLociWithSnps, 17,541 probes) and cross-reactive probes (26,569 probes) were also removed.^57^ Batch correction was performed using ComBat for array (72 batches) and subsequently chip position (12 batches). Processing steps were visualized in ShinyMethyl interface.^58^ There were no sex mismatches. Forty technical replicates were used across different clips and runs. Technical variation was assessed using MDS plots and intraclass correlation of the top 1000 most variable methylation probes. Technical replicates were removed before downstream analyses.

#### DNA Methylation and Risk of Disease Recurrence Following Surgery in Patients With CD

The composite clinical outcome used in the original TOPPIC trial consisting of an increase in Crohn’s disease activity index of more than 150 and an increase of 100 points from baseline measurement together with the institution of immunosuppressive treatment, or further surgery. Secondary outcomes of CD disease recurrence included the highest endoscopic scores (CDEIS, Rutgeerts) measured at 49 and 157 weeks following randomization. TOPPIC data alone were read into R and processed using the previously mentioned steps. DMP analysis (recurrence vs no recurrence) was performed as mentioned with the following covariates: age, sex, smoking status, treatment/placebo, and cell proportions. DVPs were assessed using the iEVORA package using the row_ievora() function in the matrixTests package with default parameter of a raw *t*-test threshold of *P* < .05 and FDR corrected *P* threshold of Bartlett test step <0.001.^28^ To adjust for covariates, a matrix of the residual values from a linear model of the covariates (age, gender, smoking status, cell proportions) was used as the input for the DVP iEVORA method. Data were submitted to the DNA methylation Clock Foundation (https://dnamage.clockfoundation.org/) for estimation of epigenetic age scores using methods by Horvath,^37^ Hannum,^38^ phenoAge,^39^ tissue specific (skin and blood clock),^40^ and GRIMage.^41^ Correlation was made with actual biologic age and estimates of age acceleration were made (methylation age – biologic age). Smoking-associated probes (DMPs) were identified using a linear model of smoking as the outcome (current vs exsmoker/never smoked) with cell proportions as covariates. Smoking-associated probes were correlated with previously published smoking-related probes.^29^^,^^59^

### Genotype and meQTL Analysis

Genotypes were called by GenomeStudio and data were processed using plink.^60^ Data assessed for sex mismatches. meQTLs were identified using the matrixEQTL package.^61^ meQTLs were identified using significant DMP and DVP methylation probes using the modelLinear function with age, sex, and smoking status as covariates to identify meQTLs (MAF >0.1, cis distance of 1 × 10^6^, min *P* value 1 × 10^6^) *P* values were FDR corrected. For disease-specific meQTLs the modelLinearCross function was used including only significant DMP and DVP methylation probes with the following covariates (age, sex, smoking status) to identify meQTLs associated with disease recurrence in the entire TOPPIC dataset (MAF 0.1, cis distance of 1 × 10^6^, min *P* value 1 × 10^6^).

### Validation of DNA Methylation Changes in IBD Cases and Control Subjects

Raw 450K HumanMethylation iDats from IBD BIOM and TOPPIC cohorts were read into R using minfi and both datasets were normalized together using the previously mentioned steps. Batch correction was performed using ComBat for array (72 batches) and chip position (12 batches).^62^^,^^63^ DMP analysis was performed using limma comparing CD cases (BIOM and TOPPIC separately) with control subjects (BIOM only^9^).^64^ The 2 CD cohorts (BIOM, TOPPIC) were analyzed together against control subjects (BIOM only) (Figure 1). The following covariates were used in linear models (age, sex, smoking status, cell deconvolution values).^65^ Correction for multiple testing was performed using the Holm adjusted *P* value.^66^ Overlap with previously published DMP lists was assessed for overrepresentation using phyper test for hypergeometric distribution.^67^ DMR analysis was performed using DMRcate with an FDR threshold of *P* < .001, Gaussian Kernel Bandwidth lamda of 500, and scaling factor C of 5.^68^^,^^69^ DVP analysis was performed using the residual matrix of a linear model of covariates (1∼ age + sex + smoking status + cell counts) with the iEVORA algorithm using the row_ievora() function in the matrixTests package with default parameter of a raw *t*-test threshold of *P* < .05 and FDR corrected *P* threshold of Bartlett test step <0.001.^39^
